# Supplementary material for: MGMT-Methylation in Non-Neoplastic Diseases of the Central Nervous System
Source: Int J Mol Sci. 2021 Apr 8;22(8):3845. doi: 10.3390/ijms22083845 (PMC8068191; doi:10.3390/ijms22083845)
Supplement: Supplementary file 1 [file ijms-22-03845-s001.pdf]

Supplementary information:
MGMT-methylation in non-neoplastic diseases of the central nervous system

Supplementary Figures:

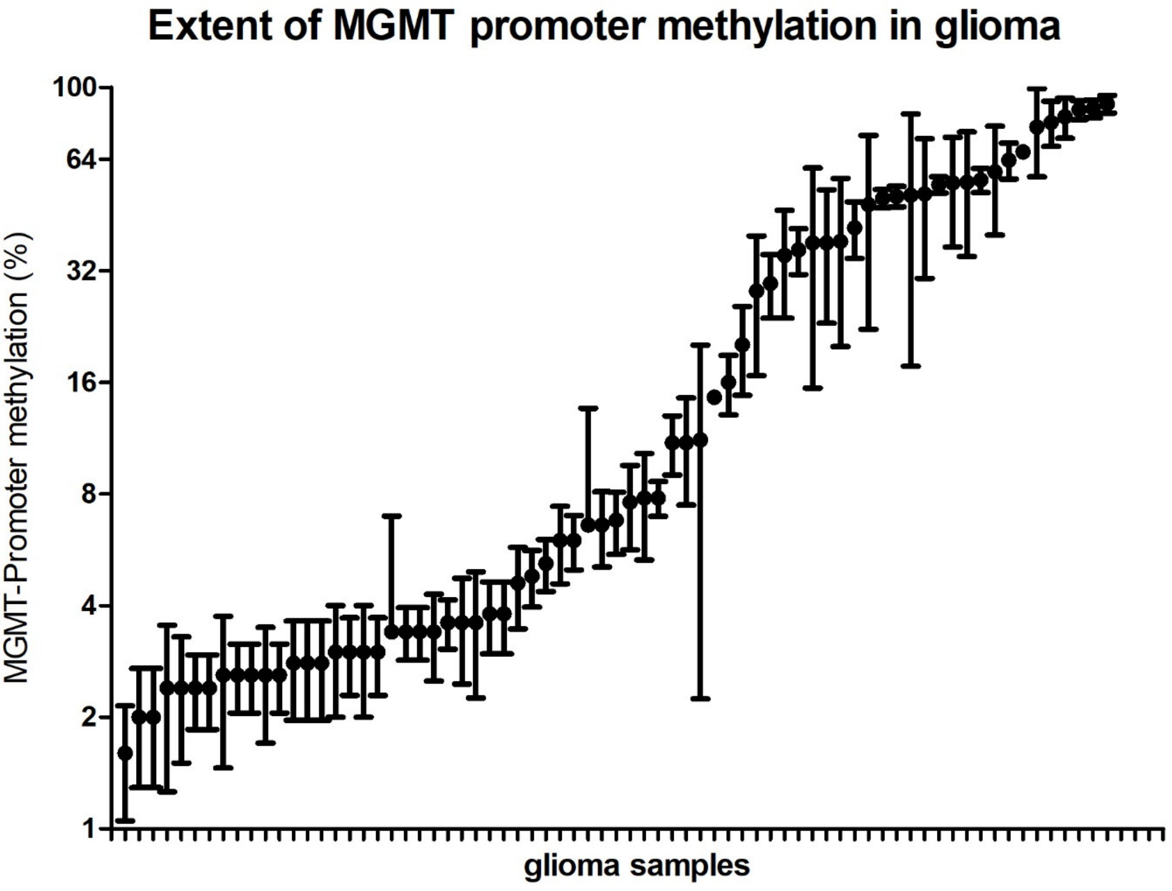

Supplementary Figure S1: Extent of MGMT promoter methylation in glioma, highlighting the broad spectrum of MGMT promoter methylation also in glioma, including cases with very low methylation rates with means of 2-3% as well as highly methylated cases with means of >90%.

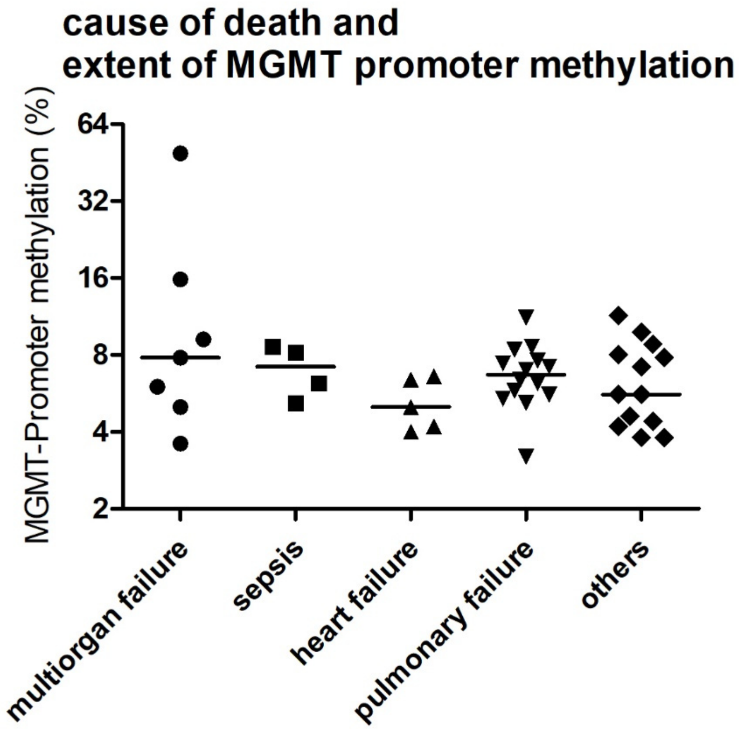

Supplementary Figure S2: Influence of cause of death on the extent of MGMT promoter methylation. In order to rule out any influence of cause of death on the extent of methylation, we categorized our autopsy samples into five groups concerning their cause of death, namely 1) multiorgan failure (n=7), 2) sepsis (n=4), 3) cardiac reasons (n=5), 4) pulmonary reasons (n=14) and 5) others (n=13). The means of methylation of those groups were compared to each other via ANOVA afterwards. We did not find any significant differences between the groups ($p>0.05$). Therefore, the (reason of) death itself does not seem to affect MGMT methylation.

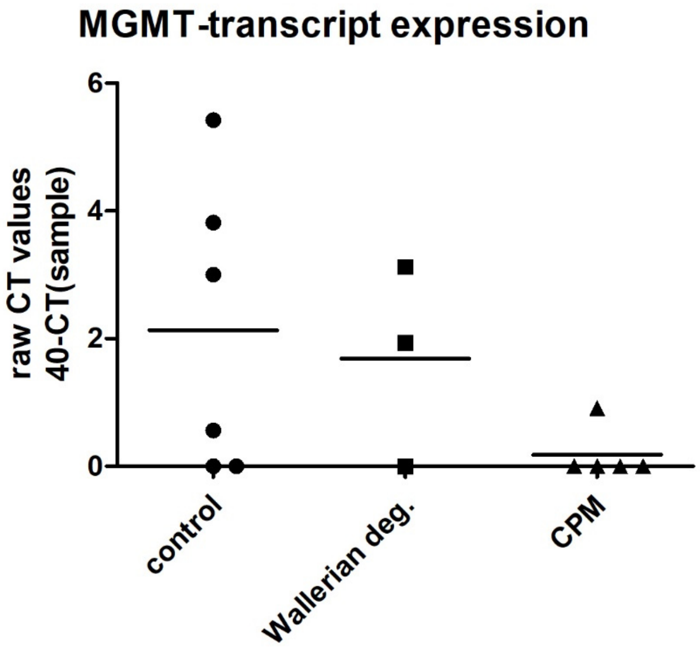

Supplementary Figure S3: MGMT mRNA expression in different CNS lesions. For quantifying MGMT transcript levels in formalin fixed paraffin embedded (FFPE) samples from healthy controls and those with hypermethylation we used qPCR (Taqman Assays). Since RNA of the lesions was almost completely degraded due to formalin fixation, the low measurable expression was mapped as raw CT values [40-CT(sample)]. Suitable results were only obtained for a few samples (healthy control (n=4); Wallerian degeneration (n=2); CPM / EPM (n=1)), and a reliable statistical assessment was not possible due to the small sample size. Nonetheless, MGMT mRNA levels tended to be lower in hypermethylated samples compared with healthy controls (Wallerian degeneration: 1.36-fold lower levels, CPM / EPM: 4-fold lower levels).

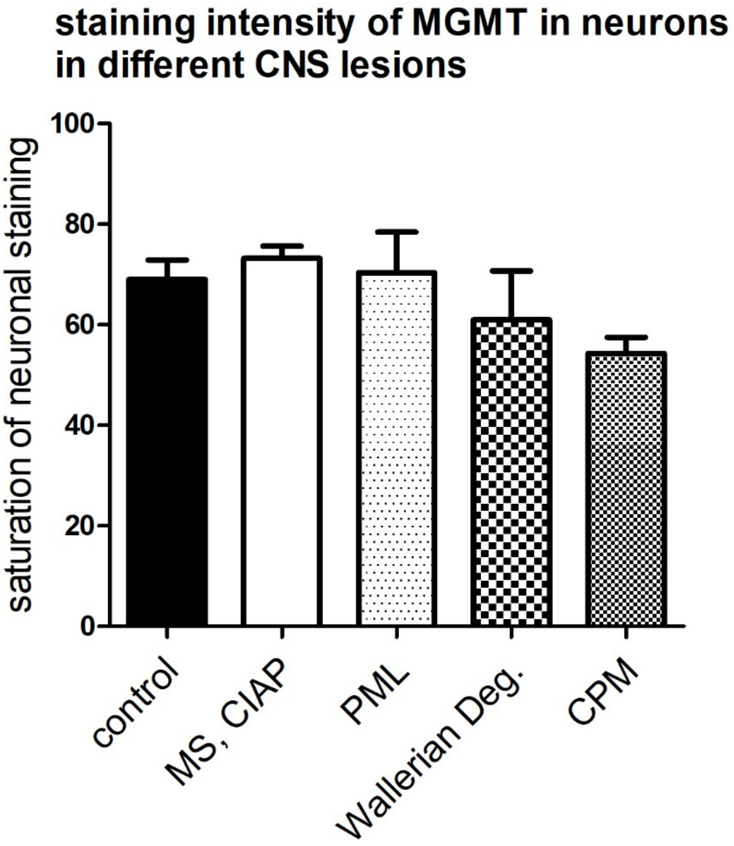

Supplementary Figure S4: Staining intensity of MGMT in neurons in different CNS lesions.
The quantification of MGMT expression in neurons was difficult to be performed. This is due to the fact, that we have analyzed samples which are located in different brain areas and thus the examined neurons show a) different morphologies, which makes it quite difficult for PC algorithms to detect all of them and b) different brain areas (e.g. cerebral cortex and pontine areas) display different neuronal cell densities, which distorts comparability of those brain areas. Therefore, it was not possible to perform a quantification of MGMT-expressing neuronal cells per mm^2 as we did in glial cells (Figure 2). As we have seen, that >90% of all neuronal cells are stained positively for MGMT in all our samples, and since staining intensities (as a marker of height of MGMT expression) varied markedly between different slides and even different areas on single slides (a phenomenon which has been referred to in the literature before [1]), we decided to investigate whether the staining intensities of MGMT in neurons differed significantly between neurons of different pathologic conditions. For the quantification of neurons, five individual neurons representative for the whole slide were selected and the saturation of color was determined in each neuron. Each pixel in an image selection has a distinct color saturation. The average saturation value of all pixels was determined by increasing the saturation level stepwise (in 256 single steps). The level below which at least 50% of all pixels had a lower saturation was defined as the average saturation level of a neuron. We did not find any significant differences between control tissues and samples of different pathologies.

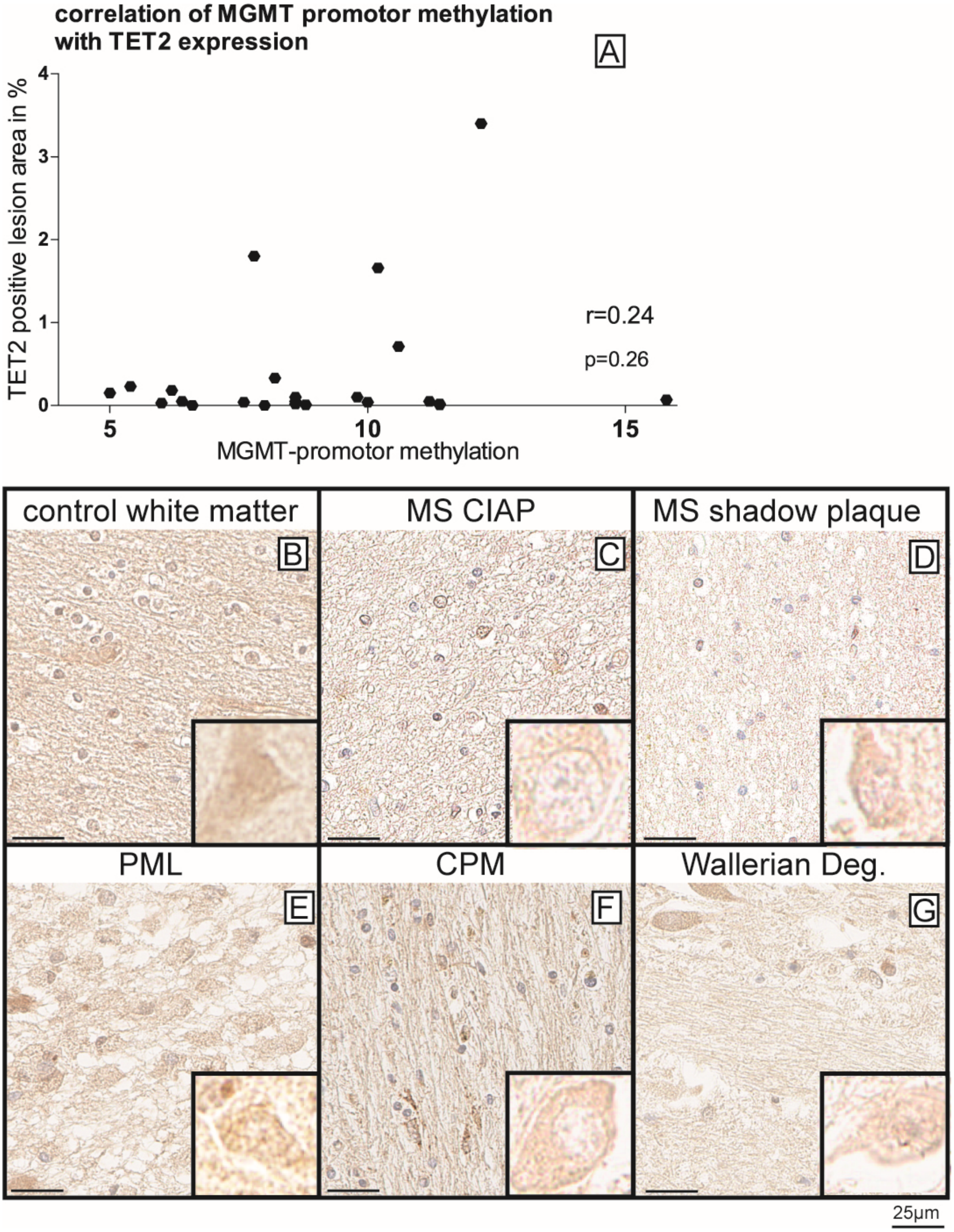

Supplementary Figure S5: TET2 staining of controls and samples with enhanced MGMT promoter methylation. There is no significant correlation between TET2 expression and MGMT promoter methylation (A). No significant differences in the immunohistochemical staining intensity were detected in controls (B), MS - chronic inactive plaque (C), MS - shadow plaque (D), PML (E), CPM (F), or Wallerian degeneration (G), neither in glial cells, nor in neurons (insets in B-G; edge length 15 µm each). Scale bar = 25µm.

Supplementary Tables:

Supplementary Table S1. MGMT promoter methylation of the five measured CpG sites in the MGMT promoter of 71 gliomas.

	Case					MGMT promoter methylation (%) at different positions					
	age	sex	cause of death	disease duration	biopsy / autopsy	pos. 1	pos. 2	pos. 3	pos. 4	pos. 5	mean (1-5)
**glioma**											
glioma 1	24	m	na	na	biopsy	7	8	5	5	5	6
glioma 2	24	f	na	na	biopsy	9	9	6	9	5	7,6
glioma 3	64	m	na	na	biopsy	85	85	84	88	96	87,6
glioma 4	23	m	na	na	biopsy	55	64	70	58	71	63,6
glioma 5	79	m	na	na	biopsy	0	3	6	0	8	3,4
glioma 6	46	m	na	na	biopsy	94	95	89	48	65	78,2
glioma 7	61	f	na	na	biopsy	3	3	4	3	4	3,4
glioma 8	68	f	na	na	biopsy	66	65	68	67	69	67
glioma 9	76	m	na	na	biopsy	1	2	2	2	3	2
glioma 10	68	m	na	na	biopsy	88	87	81	85	61	80,4
glioma 11	54	f	na	na	biopsy	76	76	15	75	14	51,2
glioma 12	71	f	na	na	biopsy	1	2	2	1	2	1,6
glioma 13	59	m	na	na	biopsy	88	65	89	89	85	83,2
glioma 14	64	f	na	na	biopsy	1	3	4	2	3	2,6
glioma 15	85	m	na	na	biopsy	2	3	3	2	3	2,6
glioma 16	79	m	na	na	biopsy	46	46	43	45	29	41,8
glioma 17	58	f	na	na	biopsy	2	3	4	2	4	3
glioma 18	63	f	na	na	biopsy	37	34	26	28	23	29,6
glioma 19	82	m	na	na	biopsy	4	4	3	3	5	3,8
glioma 20	57	m	na	na	biopsy	2	3	4	3	3	3
glioma 21	75	f	na	na	biopsy	2	2	4	3	3	2,8
glioma 22	67	f	na	na	biopsy	54	52	55	57	63	56,2
glioma 23	85	f	na	na	biopsy	4	3	4	3	4	3,6
glioma 24	83	m	na	na	biopsy	4	4	3	3	3	3,4
glioma 25	51	f	na	na	biopsy	23	16	23	26	13	20,2
glioma 26	80	m	na	na	biopsy	19	2	5	4	3	6,6
glioma 27	41	f	na	na	biopsy	12	7	6	8	6	7,8
glioma 28	72	f	na	na	biopsy	24	23	18	60	65	38
glioma 29	58	f	na	na	biopsy	37	83	30	68	23	48,2
glioma 30	79	m	na	na	biopsy	85	84	84	86	96	87
glioma 31	87	f	na	na	biopsy	2	4	4	2	3	3
glioma 32	83	m	na	na	biopsy	8	12	10	13	12	11
glioma 33	54	f	na	na	biopsy	2	3	4	2	1	2,4
glioma 34	80	f	na	na	biopsy	4	6	5	5	6	5,2
glioma 35	80	m	na	na	biopsy	1	3	3	2	3	2,4
glioma 36	41	m	na	na	biopsy	4	4	6	5	5	4,8
glioma 37	35	f	na	na	biopsy	29	42	37	40	34	36,4
glioma 38	77	m	na	na	biopsy	5	7	7	6	5	6
glioma 39	29	f	na	na	biopsy	66	64	62	66	19	55,4
glioma 40	56	m	na	na	biopsy	49	51	46	52	53	50,2
glioma 41	67	f	na	na	biopsy	2	3	3	2	3	2,6
glioma 42	71	f	na	na	biopsy	54	52	53	55	59	54,6
glioma 43	79	m	na	na	biopsy	88	88	87	89	99	90,2
glioma 44	52	m	na	na	biopsy	2	4	4	3	4	3,4
glioma 45	59	m	na	na	biopsy	14	14	15	15	15	14,6
glioma 46	59	m	na	na	biopsy	61	59	14	62	62	51,6
glioma 47	47	m	na	na	biopsy	17	20	12	15	16	16
glioma 48	49	m	na	na	biopsy	51	48	48	51	56	50,8
glioma 49	60	m	na	na	biopsy	2	2	3	2	3	2,4
glioma 50	74	m	na	na	biopsy	2	3	4	3	3	3
glioma 51	20	f	na	na	biopsy	3	4	4	3	5	3,8
glioma 52	33	m	na	na	biopsy	16	13	9	10	7	11
glioma 53	84	m	na	na	biopsy	2	2	3	2	4	2,6
glioma 54	80	f	na	na	biopsy	2	3	4	2	3	2,8
glioma 55	55	f	na	na	biopsy	81	64	48	50	33	55,2
glioma 56	65	m	na	na	biopsy	5	9	8	7	27	11,2
glioma 57	41	f	na	na	biopsy	7	9	8	8	7	7,8
glioma 58	70	f	na	na	biopsy	32	47	47	22	28	35,2
glioma 59	51	m	na	na	biopsy	5	8	7	8	6	6,8
glioma 60	69	m	na	na	biopsy	2	2	3	2	3	2,4
glioma 61	66	f	na	na	biopsy	47	48	29	16	50	38
glioma 62	66	m	na	na	biopsy	2	4	4	3	5	3,6
glioma 63	65	f	na	na	biopsy	2	3	3	2	3	2,6
glioma 64	70	m	na	na	biopsy	1	2	3	2	2	2
glioma 65	56	m	na	na	biopsy	33	32	17	43	16	28,2

glioma 66	76	m	na	na	biopsy	2	3	4	2	3	2,8
glioma 67	63	m	na	na	biopsy	71	53	70	28	74	59,2
glioma 68	66	f	na	na	biopsy	3	5	5	4	6	4,6
glioma 69	81	m	na	na	biopsy	4	7	7	7	8	6,6
glioma 70	81	m	na	na	biopsy	2	3	5	3	5	3,6
glioma 71	26	f	na	na	biopsy	12	50	52	52	26	38,4

Abbreviations: m, male; f, female; na, not applicable

Supplementary Table S2: Correlations of MGMT promoter methylation with clinical and immunohistochemical parameters.

**clinical or immunohistochemical parameter**	**parameter value**	**Pearson's correlation coefficient r**	**p-Value**
age	51.8y+-15.8	0.01	0.93
sex	f(33), m(41)	0.07	0.57
number of apoptotic cells per mm² (caspase-3 staining)	14.76+-9.24	0.23	0.24
Density of phosphorylated neurofilaments (SMI31 staining)	71.5%+-20.33	0.05	0.78

Supplementary References:

1. Hsu, C. Y.; Lin, S. C.; Ho, H. L.; Chang-Chien, Y. C.; Hsu, S. P.; Yen, Y. S.; Chen, M. H.; Guo, W. Y.; Ho, D. M., Exclusion of histiocytes/endothelial cells and using endothelial cells as internal reference are crucial for interpretation of MGMT immunohistochemistry in glioblastoma. *Am J Surg Pathol* **2013,** 37, (2), 264-71.
